# Approach of Supercritical Carbon Dioxide for the Extraction of Kleeb Bua Daeng Formula

**DOI:** 10.3390/molecules28196873

**Published:** 2023-09-29

**Authors:** Nittaya Ngamkhae, Orawan Monthakantirat, Yaowared Chulikhit, Juthamart Maneenet, Charinya Khamphukdee, Yutthana Chotritthirong, Suphatson Limsakul, Chantana Boonyarat, Supaporn Pitiporn, Pakakrong Kwankhao, Anake Kijjoa, Supawadee Daodee

**Affiliations:** 1 Division of Pharmaceutical Chemistry, Faculty of Pharmaceutical Sciences, Khon Kaen University, Khon Kaen 40002, Thailand; nittaya.ng@kkumail.com (N.N.); oramon@kku.ac.th (O.M.); yaosum@kku.ac.th (Y.C.); juthamart_pp@hotmail.com (J.M.); yutthana_ch@kkumail.com (Y.C.); suphatson.l@kkumail.com (S.L.); chaboo@kku.ac.th (C.B.); ankijjoa@icbas.up.pt (A.K.); 2 Division of Pharmacognosy and Toxicology, Faculty of Pharmaceutical Sciences, Khon Kaen University, Khon Kaen 40002, Thailand; charkh@kku.ac.th; 3 Department of Pharmacy, Chao Phraya Abhaibhubejhr Hospital, Ministry of Public Health, Prachinburi 25000, Thailand; spitiporn@yahoo.com (S.P.); pakakrong2@gmail.com (P.K.); 4 Instituto de Ciências Biomédicas Abel Salazar and CIIMAR, Universidade do Porto, Rua Jorge de Viterbo Ferreira 282, 4050-313 Porto, Portugal

**Keywords:** supercritical fluid extraction, response surface methodology, Box–Behnken design, Kleeb Bua Daeng formula

## Abstract

Supercritical fluid extraction (SFE) is an innovative green technology for the extraction of phytochemicals from plants. Therefore, this study aimed to evaluate the application of SFE and to optimize the extraction conditions of the Thai herbal formula, Kleeb Bua Daeng (KBD). A Box–Behnken design (BBD) with response surface methodology (RMS) was used to determine the effect of the extraction time (30–90 min), temperature (30–60 °C), and pressure (200–300 bar) on response variables including the extraction yield, total phenolic content (TPC), total flavonoid content (TFC), total carotenoid content (TCC), and total anthocyanin content (TAC) of the KBD formula. The highest percentage extraction yield (3.81%) was achieved at 60 °C, 300 bar, and 60 min of the extraction time. The highest TPC (464.56 mg gallic acid equivalents/g extract), TFC (217.19 mg quercetin equivalents/g extract), and TCC (22.26 mg β-carotene equivalents/g extract) were all achieved at 60 °C, 250 bar, and 90 min of the extraction time. On the contrary, it was not possible to quantify the total anthocyanin content as anthocyanins were not extracted by this method. The results indicated that SFE-CO_2_ is a suitable method of extraction for a green recovery of phytochemicals with low and moderate polarity from the KBD formula.

## 1. Introduction

Kleeb Bua Daeng (KBD) is a Thai traditional herbal formula developed by Chao Phraya Abhaibhubejhr hospital in Prachinburi Province, Thailand. Many phytochemicals such as phenolics, flavonoids, and carotenoids have been found in this formula. KBD is used for treatment of insomnia and to improve memory. Safety and efficacy of this formula in mild cognitive impairment symptoms were reported. KBD was also found to improve the cognitive impairment in an unpredictable chronic mild stress mice model [1,2]. KBD consists of three herbs: *Nelumbo nucifera* (petals)*, Piper nigrum* (fruits), and *Centella asiatica* (aerial part). The petals of *N. nucifera* were found to contain many bioactive compounds with antioxidant, anti-inflammatory, neuroprotective, and anticancer activities [3,4]. In turn, the fruits of *P. nigrum* or black pepper, which display anti-inflammatory, antioxidant, anticancer, antidepressant, and analgesic activities [5,6], were found to contain the alkaloid piperine as a major constituent, in addition to polyphenols and flavonoids [7,8], while *C. asiatica*, which has antioxidant, antiulcer, antidepressant, and anti-inflammatory activities [9,10], contains asiaticoside, polyphenols, flavonoids, and carotenoids [11].

Extraction is the most important step in isolating bioactive compounds from plants and other biological materials. Various traditional extraction techniques such as heat reflux, maceration, and Soxhlet extraction normally require a large amount of solvent and a long extraction time. As new extraction technologies have emerged, advanced extraction methods, including ultrasound-assisted extraction (UAE), microwave-assisted extraction (MAE), pulsed electric field-assisted extraction, pressurized liquid extraction, enzyme-assisted extraction, and supercritical fluid extraction, have recently been developed [12,13,14]. These modern extraction techniques provide fast and effective extraction with less amount of solvent used.

Supercritical fluid extraction (SFE) is a technique widely used to extract natural products and usually employs non-toxic solvents such as carbon dioxide (CO_2_), water, nitrous oxide, and ethane. SFE is carried out at pressures exceeding the critical pressure of the extracting solvent. This choice of high pressure is made because it enhances the solvent’s density and diffusion coefficient. Furthermore, even slight adjustments in pressure and temperature can significantly influence the extraction selectivity [15]. SFE allows a separation of the extracting solvent from the extract simply by the expansion of the fluid in the extractor vessel outlet with a pressure drop, causing the fluid to change to a gas phase, and thus separate from the solid that it extracts. CO_2_ is the most commonly used extraction solvent in SFE. Supercritical carbon dioxide (ScCO_2_; critical point: 7.38 MPa, 304 K/31.1 °C, and 73.8 bar) is a nonpolar medium with a large quadrupole moment. Its density can be changed as a function of temperature and pressure. At a critical pressure, its compressibility is maximized, and small changes in parameters can lead to large changes in its local density [16]. The advantages of CO_2_ are that it is non-flammable, non-toxic, non-explosive, economical, and easily removable from the extract. Many parameters such as the temperature, pressure, particle size, solvent-to-feed ratio, extraction time, and flow rate of CO_2_ can influence the extraction efficiency [17]. Thus, optimization of these parameters could be performed to increase the percentage yield, the content of active ingredients, and the biological activity of the extract.

The Box–Behnken Design (BBD) and response surface methodology (RSM) are the statistical analysis method and simple experimental design tools for the effective optimization of the extraction process. These tools are easy to use and provide good results. A suitable number of experimental runs from the BBD provided a higher advantage when compared with other statistical designs [18]. SFE involves many variables which may affect the efficiency of extraction. The selection of critical variables and their levels is important. RSM has previously been applied for the optimization of SFE for the extraction of bioactive compounds from many plants. For example, optimization of SFE with RSM was used for the extraction of essential oils from orange (*Citrus sinensis*) peel [19] as well as the extraction of polyphenols from grape pomace [20].

The novelty of this work is twofold. First, this is the first application of SFE extraction by using ScCO_2_ to extract bioactive compounds from the traditional Thai herbal formula, KBD, which is a mixture of different parts of three plants, thus consisting of different types of compounds with different physico-chemical properties. This study is in contrast to other previous studies that used SFE with CO_2_ to extract compounds, normally belonging to the same chemical families, of only one plant material. Moreover, KBD is normally extracted by traditional processes such as SLE, and to a lesser extent, by more modern techniques such as MAE and UAE. Therefore, this study represents the development of an effective green technology extraction process that is not only eco-friendly but also guarantees safety for extracts of medicinal products by using a non-toxic extracting solvent that is completely eliminated such as CO_2_.

Thus, the objective of this study was to assess the conditions for SFE using ScCO_2_ to optimize the extraction of the total active compounds from KBD, a process not previously explored. The study involved a variation of several extraction parameters, including extraction time, pressure, and temperature, using the BBD with RSM to determine the appropriate extraction conditions to achieve the highest percentage of extraction as well as the maximum contents of active compounds. This study can be applied to give a new prospect to obtain a high-quality extract for product development.

## 2. Results

### 2.1. Optimization of Variable Conditions for SFE

The BBD and RSM were used to optimize the SFE conditions. Three extraction variables, i.e., extraction temperature, extraction pressure, and extraction time, and six responses including percentage extraction yield, total flavonoid content (TFC), total phenolic content (TPC), total carotenoid content (TCC), total anthocyanin content (TAC), and percentage inhibition of the DPPH assay were evaluated in this study. ScCO_2_ was chosen as an extraction solvent because it is safe and non-toxic compared to other organic solvents. From the analysis of variance (ANOVA), the quadratic polynomial model was highly significant with the *p*-value less than 0.0001 for TCC, 0.0265, 0.0162, and 0.0047 for percentage extraction yield, TPC, and TFC, respectively. The experimental results of 17 runs and their predicted values from the equation model of each response are shown in Table 1.

### 2.2. Effects of SFE Conditions on the Percentage Extraction Yield

The quadratic model for the percentage extraction yield of KBD powder showed significant linear and quadratic effects, as shown by Equation (1).

Y_Yield_ = +3.49 + 0.1738A + 0.2588B + 0.0975C − 0.0400AB − 0.0375AC − 0.1125BC + 0.0665A^2^ − 0.0735B^2^ − 0.2060C^2^ (1)

where Y is the response variable (percentage extraction yield), A is the extraction temperature, B is the extraction pressure, and C is the extraction time. A, B, and C are linear terms of each factor; AB, AC, and BC are interaction terms among factors; and A^2^, B^2^, and C^2^ are quadratic terms of factors.

As shown in Table 2, the model was significant at a *p*-value < 0.05. A model F-value of 4.72 was obtained, implying that the model was significant. The coefficient of determination (R^2^) and the adjusted coefficient of determination (Adj.R^2^) were 0.8584 and 0.6764, respectively. In this case, A, B, and C^2^ were significant model terms due to the high estimated coefficient from the model equation (Equation (1)). The experimental results showed the extraction yield was between 2.67 and 3.81%. No interaction effect to the percentage extraction yield among factors was found, i.e., the *p*-value was more than 0.05 for all interaction terms, AB, AC, and BC. The highest percentage extraction yield (3.81%) was found at 60 °C of the extraction temperature, 300 bar of the extraction pressure, and 60 min of the extraction time. Higher temperature and pressure seemed to furnish the higher percentage extraction yield.

### 2.3. Effects of SFE Conditions on Total Phenolic Content (TPC)

The linear-quadratic model for TPC in KBD powder showed significant linearity at a *p*-value less than 0.05 and an F-value of 5.66. All quadratic effects are shown in Equation (2).

Y_TPC_ = +155.38 + 77.38A + 17.22B + 52.52C + 20.00AB + 84.36AC − 87.83BC + 33.82A^2^ + 17.02B^2^ + 22.32C^2^ (2)

From the ANOVA analysis, the quadratic model of TPC was fitted significantly (*p*-value < 0.05) and revealed R^2^ = 0.8792 and Adj.R^2^ = 0.7239. The high coefficient of a particular factor in the equation indicated a strong impact on the response variable, TPC. In this case, the extraction temperature (A), the extraction time (C), the interaction effect between extraction temperature and time (AC), and the interaction effect between the extraction pressure and time (BC) were significant model terms (*p*-value < 0.05) and were most significant parameters affecting the TPC.

**Figure 1:**
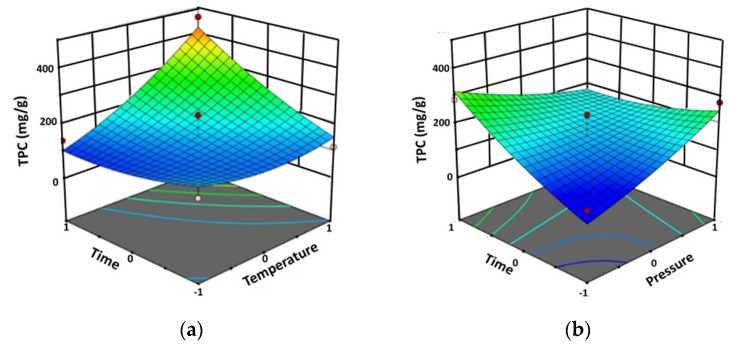
Three-dimensional response surface plots of TPC by Design-Expert software using SFE method against significant interaction factors: (**a**) between the extraction temperature and the extraction time (AC), and (**b**) between the extraction pressure and the extraction time (BC).

The results showed that the TPC was between 82.33 and 464.56 mg gallic acid equivalents/g extract (mg GAE/g extract). The highest TPC (464.56 mg GAE/g extract) was determined at the extraction temperature of 60 °C, the extraction pressure of 250 bar, and the extraction time of 90 min. Figure 1 shows the interaction effect between the extraction temperature and the extraction time (AC) and between the extraction pressure and the extraction time (BC) on the TPC.

### 2.4. Effects of SFE Conditions on Total Flavonoid Content (TFC)

The TFC in KBD powder was investigated and the quadratic model showed significant linear and quadratic effects according to Equation (3).

Y_TFC_ = +165.46 − 3.15A + 11.21B + 8.75C + 5.18AB + 2.78AC + 3.94BC + 24.26A^2^ − 18.18B^2^ + 10.64C^2^ (3)

From the ANOVA result in Table 2, the model F-value of 8.67 implied this model is significant and the *p*-value of this quadratic model was 0.0047. The R^2^ and Adj.R^2^ were 0.9177 and 0.8119, respectively. The extraction pressure and extraction time were the most significant parameters affecting the TFC. The interaction effect between factors was not significant. However, the quadratic terms of this model (A^2^, B^2^, and C^2^) significantly affect the TFC at a *p*-value less than 0.05.

The experimental results showed the TFC was between 142.08 and 217.19 mg quercetin equivalents/g extract (mg QE/g extract). The highest TFC (217.19 mg QE/g extract) was achieved at the extraction temperature of 60 °C, the extraction pressure of 250 bar, and the extraction time of 90 min.

### 2.5. Effects of SFE Conditions on Total Carotenoid Content (TCC)

The quadratic model for total carotenoid content (TCC) showed significant linear and quadratic effects according to Equation (4).

Y_TCC_ = +10.74 + 4.12A + 1.25B + 1.12C − 0.3425AB + 5.78AC − 1.38BC + 2.67A^2^ + 0.0570B^2^ − 1.79C^2^ (4)

**Figure 2:**
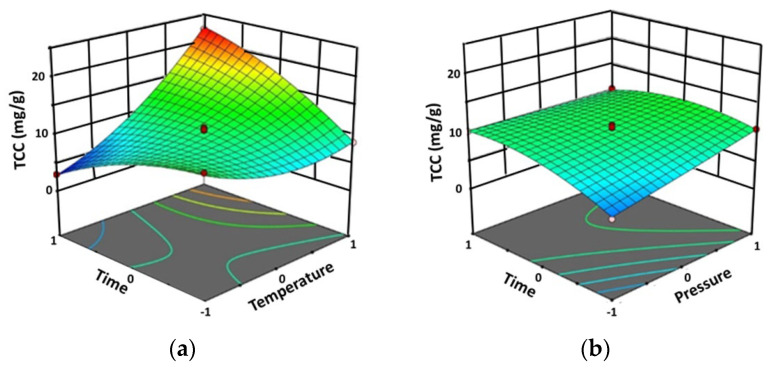
Three-dimensional response surface plots of TCC by Design-Expert software using SFE method against significant interaction effect: (**a**) between factors (the extraction temperature and the extraction time (AC) and (**b**) between the extraction pressure and the extraction time (BC).

The model showed a *p*-value < 0.0001 with a high F-value (77.70), implying that the model was significant (Table 2). In this case, the extraction temperature (A) significantly affected the TCC with a *p*-value < 0.0001. Meanwhile, the extraction pressure (B) and the extraction time were significantly affected at a *p*-value < 0.005. The interactions between the extraction temperature-time (AC) and extraction pressure-time (BC) significantly affected the TCC, as shown by the three-dimensional response surface plots (Figure 2). The quadratic term of the extraction temperature (A^2^) and extraction time (C^2^) exhibited a significant effect at a *p*-value < 0.05.

The R^2^ and Adj.R^2^ were 0.9901 and 0.9773, respectively. Since the Adj.R^2^ was close to R^2^, the model was highly significant. The results showed that the TCC was between 2.99 and 22.26 mg β-carotene equivalents/g extract (mg β-CE/g extract). The highest TCC (22.26 mg β-CE/g extract) was found with an extraction temperature of 60 °C, an extraction pressure of 250 bar, and an extraction time of 90 min.

### 2.6. Effects of SFE Conditions on Total Anthocyanin Content (TAC)

The results from the experiments to determine the TAC revealed that anthocyanin could not be detected in this study even though the conditions of important factors were varied. That means anthocyanin could not be extracted by this method. The polarity of supercritical CO_2_ fluid might not be appropriate for the extraction of less soluble compounds from the KBD powder. Some studies have described the use of a co-solvent such as methanol, ethanol, acetic acid, and formic acid for enhancing the efficiency of extraction [21]. However, ethanol seems to be the preferred co-solvent for consumable products due to its low toxicity. Some polar or nonpolar co-solvents can be used for SFE to improve the solvation of ScCO_2_ which can enhance the affinity of poorly soluble compounds.

## 3. Discussion

We have previously used the BBD with RSM to determine the type of solvents, extraction time, and ratio between the material and solvent for the conventional solid–liquid extraction (SLE) [1], and the effect of extraction time, temperature, solvent concentration, and material-to-solvent ratio for the UAE and MAE of KBD powder [22]. A comparison of the results obtained in separate experiments by UAE and MAE showed that MAE furnished a higher percentage extraction yield, total flavonoid content (TFC), total phenolic content (TPC), total carotenoid content (TCC), and total anthocyanin content (TAC) than UAE [22].

Due to the need for an eco-friendly and green technology of extraction, we have used the BBD with RSM to design the SFE, with ScCO_2_ as a solvent, for the extraction of chemical constituents of KBD powder. By changing the three variables, i.e., extraction temperature, extraction pressure, and extraction time, the percentage of extraction yield, TFC, TPC, and TCC can be optimized.

The model showed that the percentage of extraction yield depends on the linear effects of the three variables viz. extraction temperature (A), extraction pressure (B), and extraction time (C), as well as on the quadratic effect of extraction time (C^2^). However, the percentage extraction yield is not affected by the interaction between the variables, i.e., the interactions between the extraction temperature and pressure (AB), the extraction temperature and the extraction time (AC), and the extraction pressure and the extraction time (BC). Table 1 shows the experimental and predicted values of the percentage yields of 17 runs with varying extraction temperature (A), extraction pressure (B), and extraction time (C). From Table 1, it was found that the minimum percentage yield is 2.67% (predicted 2.74%, Δ = −0.13%) when the extraction was performed at 45 °C and 200 bar during 30 min of extraction, while the maximum yield is 3.81% (predicted 3.88%, Δ = −0.07%) when the extraction was performed at 60 °C and 300 bar during 60 min of extraction. The results displayed in Table 1 show that the increase in temperature, pressure, and extraction time resulted in an increase in the percentage extraction yield.

On the other hand, the extraction temperature (A) and the extraction time (C), as well as the interactions between the extraction temperature and the extraction time (AC) and between the extraction pressure and the extraction time (BC) exert a strong effect on the TPC. The minimum TPC of 82.33 mg GAE/g extract (predicted 37.15 mg GAE/g extract, Δ = +45.18 GAE/g extract) was obtained at 45 °C and 200 bar during 30 min of extraction time (the same conditions that gave the minimum percentage yield), while the maximum TPC of 464.56 mg GAE/g extract (predicted 425.78 mg GAE/g extract, Δ = +38.78 GAE/g extract) was achieved at 60 °C and 250 bar during 90 min of extraction (Table 1). The results shown in Table 2 reveal that the interaction between the extraction temperature and the extraction time (AC), as well as between the extraction pressure and the extraction time, (BC) affected the TPC (Figure 1).

In turn, the extraction pressure and extraction time, as well as the quadratic terms of extraction temperature, pressure, and time (A^2^, B^2^, and C^2^) showed significant effects on the TFC, while the interactions between these parameters did not show any significant effect on TFC. Table 1 shows the minimum TPC of 142.08 mg QE/g extract was obtained at 60 °C and 200 bar during 60 min of extraction time (predicted 151.99 QE/g extract, Δ = −9.91 QE/g extract) while the maximum TFC of 217.19 mg QE/g extract (predicted 208.74 mg QE/g extract, Δ = +8.45 QE/g extract) was achieved at 60 °C and 250 bar during 90 min of extraction time.

Finally, it was found that the extraction temperature (A) showed a great effect on the TCC while the extraction pressure (B) and the extraction time (C) also exerted their effects, but to a lesser extent. Figure 2 shows that the TCC was also significantly affected by the interactions between extraction temperature and extraction time (AC) as well as between extraction pressure and extraction time (BC). Table 1 shows that the minimum TCC of 2.99 mg β-CE/g extract (predicted 2.86 mg β-CE/g extract, Δ = +0.13 mg β-CE/g extract) was obtained at 30 °C and 250 bar during 90 min of extraction time, while the maximum TCC of 22.26 mg β-CE/g extract (predicted 22.64 mg β-CE/g extract, Δ = −0.38 mg β-CE/g extract) was achieved in the same conditions as those for the maximum TPC and TFC, i.e., 60 °C and 250 bar during 90 min of extraction time.

**Figure 3:**
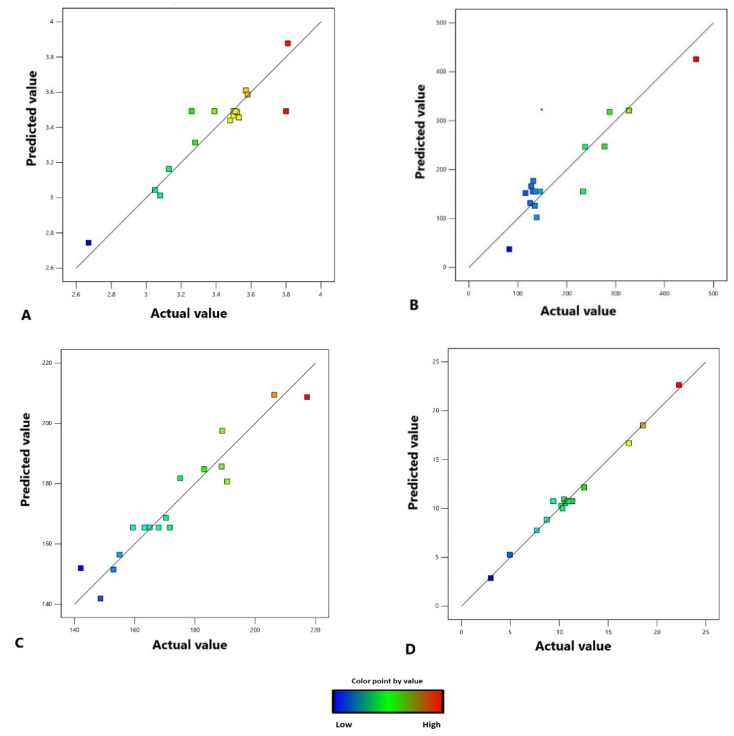
The relationship between the experimental values (actual values) and the predicted values of the percentage extraction yield (**A**), TPC (**B**), TFC (**C**), and TCC (**D**).

The results obtained from the experiments revealed that all the models obtained by the BBD with the RSM of the three variable factors in this study were significant for all the responses, i.e., percentage of extraction yield, TPC, TFC, and TCC. Consequently, these models using the three variable factors can be used for the prediction of these responses. As shown in Figure 3, there is a high correlation between the experimental and predicted values of each response, i.e., percentage yield, TPC, TFC, and TCC. The desirability approach was used to achieve the optimal balance to obtain the highest response of extraction yield, TPC, TFC, and TCC at the same time. High desirability was achieved when the extraction temperature, pressure, and extraction time were 60 °C, 271.5 bar, and 90 min, respectively. The prediction response values from this predicted condition were 3.58%, 425.78 mg GAE/g extract, 208.74 mg QE/g extract, and 22.64 β-CE/g extract, respectively.

SFE, using ScCO_2_ as a solvent, showed a good potential for the extraction of flavonoids and carotenoids. However, since ScCO_2_ assumes the characteristics of a nonpolar solvent (it is comparable to n-hexane), it has some inherent limitations since the extractability of the compounds depends on their chemical structures as well as their polarity and molecular weight [21]. Some polar compounds have limited solubility in ScCO_2_ [23,24]. Some studies revealed that hydrocarbon and other organic compounds, with low polarity and a molecular weight of less than 250, can exhibit excellent solubility in ScCO_2_ which can be carried out at low pressure (75–100 bar). Compounds of moderate polarity, with a molecular weight between 250 and 400, are moderately soluble in ScCO_2_ and, therefore, higher pressure is needed for extraction. Highly polar compounds with a molecular weight higher than 400 are scarcely soluble or insoluble in ScCO_2_ and are not extractable. This is the reason why anthocyanins and their glycosides could not be extracted and quantified by SFE in this study. Anthocyanins contain many phenolic hydroxyl groups and are more soluble in polar organic solvents such as ethanol and methanol. Thus, a non-polar ScCO_2_ is not appropriate for the extraction of anthocyanins. However, SFE can be used for the extraction of anthocyanins by adding some co-solvents such as water and ethanol to increase the polarity of the extracting solvent [25].

When comparing the results of solid–liquid extraction (SLE) [1] with those of UAE and MAE [22] for the extraction of KBD powder, it was found that the percentage yield obtained by SLE was higher than those obtained by UAE but lower than that obtained by MAE. On the other hand, the TPC obtained by SLE was comparable to that obtained by UAE, but much lower than that obtained by MAE. In turn, the TFC obtained by SLE was higher than that obtained by UAE but much lower than that obtained by MAE. Interestingly, the TCC obtained by SLE was comparable to those obtained by UAE and MAE, while the TAC obtained by SLE was lower than those obtained by UAE and MAE. It is worth mentioning that SLE, UAE, and MAE were able to extract, even in a small amount, and quantify anthocyanins from KBD powder.

A comparison of the results obtained by SFE in this study with those obtained previously by UAE and MAE [22] revealed that the percentage yield obtained by SFE is much lower than that of UAE and MAE. On the other hand, the TPC obtained by SFE was comparable to that obtained by MAE but much higher than that obtained by UAE, while the TFC and TCC obtained by SFE were much higher than those obtained by MAE and UAE. Since SFE was performed in separate experiments from MAE and UAE, the results obtained from these three methods cannot be compared in absolute terms. However, the overall results showed the tendency of which methods of extraction are appropriate to extract bioactive compounds from KBD powder.

The results obtained by us coincided with those obtained by Georgiopoulou et al. who examined the effect of temperature, time, and solvent-to-biomass ratio for SLE, temperature, time, solvent-to-biomass ratio, and microwave power for MAE, and temperature, pressure, solvent flow rate, and co-solvent presence for SFE on the yield of the extract, phenolic, chlorophyll, carotenoid content, and antioxidant activity of a microalga, *Scenedesmus obliquus*. These authors have found that MAE was more efficient than SLE in all terms. On the other hand, SFE was found to give the lowest yield and chlorophyll content but higher carotenoid content and improved antioxidant activity. Moreover, the addition of a co-solvent significantly improved the yield of SFE and led to the most superior extract [26].

The same group has also used a Face-Centered Central Composite Design (FC-CCD) with RSM to optimize the process of MAE to obtain chlorophyll, carotenoid, and phenolic compounds from a microalga, *Chlorella vulgaris*, and compared the results with those obtained from SLE and SFE [27]. They have found that, under the optimized conditions, the percentage extraction yield obtained by SLE was higher than that obtained by MAE, which was, in turn, higher than that obtained by SFE. Interestingly, SFE with ScCO_2_ with 10% ethanol as a co-solvent increased the percentage extraction yield obtained by SFE using ScCO_2_ alone twofold. Interestingly, the TPC obtained by SFE with 10% ethanol and SFE co-solvent was higher than that obtained by SLE and MAE, respectively. On the other hand, the TCC showed an increasing trend from SLE, MAE, SFE to SFE-10% ethanol.

A comparative study of UAE, MAE, SFE-CO_2_, and classical methods for extraction of alkaloids from *Mitragyna speciosa* leaves also showed that MAE gave higher alkaloid yield than other techniques [28].

## 4. Materials and Methods

### 4.1. Plant Materials

The KBD used in this study is the Thai traditional herbal formula containing three herbs: *Piper nigrum* Linn. (voucher specimen ABH18), *Nelumbo nucifera* Garetn. (voucher specimen ABH15), and *Centella asiatica* Linn. (voucher specimen ABH17) and their voucher specimens were deposited at Chao Phraya Abhaibhubejhr Hospital Foundation under the Royal Patronage of H.R.H. Princess Bejraratanarajsuda, Prachinburi Province, Thailand.

### 4.2. Chemicals and Reagents

Folin-Ciocalteu phenols reagent, sodium acetate, Trolox, and quercetin were purchased from Sigma-Aldrich (St. Louis, MO, USA). Acetonitrile, methanol, and ethanol were purchased from VWR Chemicals BDH (Leicester, England). Gallic acid, phosphoric acid, and hydrochloric acid were purchased from Merck (Darmstadt, Germany). Sodium carbonate was bought from loba chemie (Mumbai, India), aluminum chloride from Ajax Finechem (New South Wales, Australia), and potassium chloride from *QRëC* (Auckland, New Zealand).

### 4.3. Experimental Design

The BBD was used to set up the SFE experimental set for response surface optimization. The BBD represents a new paradigm in experimental design. Its fusion of rotatable or nearly rotatable second-order design with a three-level incomplete factorial design creates a powerful framework for conducting experiments. The BBD is easy to perform and interpret compared to other models [29]. The experimental design was performed using Design-Expert software (Version 13, Stat-Ease Inc., Minneapolis, USA). Seventeen experimental runs were used to extract the KBD powder by SFE. The variable factors and their levels are presented in Table 3. The temperatures used in the experiments were from 30 to 60 °C. The highest temperature of 60 °C was chosen to avoid the decomposition of some bioactive compounds such as phenolic compounds [30]. The maximum extraction time was 90 min. as an extraction time longer than this is not considered useful for the extraction method.

The second-order polynomial model (quadratic model) for the response surface analysis is shown as follows:

(5) $${Y=\alpha }_{0}+\sum \alpha_{i}X_{i\;}+\sum {\;\alpha }_{\mathrm{ii}}X_{i}^{2}+\sum {\;\alpha }_{\mathrm{ij}}X_{i}X_{j}$$

where Y is the response variable, α_0_ is a constant, α_i_ is the linear effect, α_ii_ is the quadratic effect, and α_ij_ is the interaction effect, and X_i_ and X_j_ are the independent variables.

### 4.4. Supercritical Fluid Extraction (SFE) for KBD Formula

**Figure 4:**
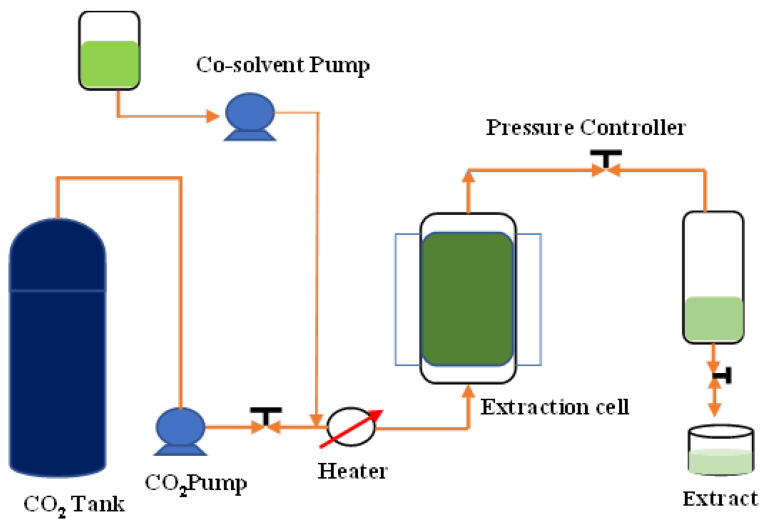
Schematic representation of supercritical fluid extraction instrument.

The ScCO_2_ extraction was performed using supercritical fluid extraction equipment (Taiwan Supercritical Technology Co., Ltd., Changhua, Taiwan) (Figure 4) and the extraction parameters are in accordance with the previously optimized method. The solvent used in the extraction was fluid CO_2_. The extraction vessel was a 1000 mL stainless steel vessel. The extractions were conducted at pressures of 200, 250, and 300 bar, temperatures of 30, 45, and 60 °C, and extraction times of 30, 60, and 90 min. KBD powder (30 g) was added into the extraction vessel and extracted with ScCO_2_ under various conditions according to the experimental design.

Table 4 shows all the experimental conditions for each of the extraction runs. After extraction, the extracts were collected and the percentage yields of the crude extract were calculated for each set of experiments. The extracts were stored at −20 °C prior to further analysis. All experiments were performed in triplicate.

### 4.5. Determination of the Extraction Yield

The extraction yield of the KBD by SFE was calculated by the following Equation (6):

Extraction yield (%) = M1/M2 × 100(6)

where M1 is the weight of KBD crude extract from SFE and M2 is the weight of the KBD dried powder.

### 4.6. Analysis of Phytochemical Content in KBD Extract

#### 4.6.1. Determination of the Total Phenolic Content (TPC)

The Folin-Ciocalteu method was used for the determination of the TPC in the KBD extract, as previously described by Ngamkhae et al. [22]. Briefly, the extract (20 μL) was mixed with 10% Folin-Ciocalteu reagent (100 μL) and 80 μL of 7% aqueous solution of Na_2_CO_3_. Then, the mixture was incubated in the dark at room temperature for 30 min. The microplate reader (PerkinElmer, Inc., Waltham, MA, USA) was used for an absorbance measurement at 760 nm. The experiments were performed in triplicate. The results are expressed as mg GAE/g extract.

#### 4.6.2. Determination of the Total Flavonoid Content (TFC)

The aluminum chloride colorimetric method was used for the determination of the TFC in the KBD extract, as previously described by Ngamkhae et al. [22]. Briefly, 20 μL of the KBD extract was added to a mixture of AlCl_3_ solution (15 μL), 10% sodium acetate (20 μL), and distilled water (20 μL). The solution was then incubated in the dark for 15 min and the absorbance was measured at 430 nm by a microplate reader (PerkinElmer, Inc., MA, USA). These experiments were conducted in triplicate, and the results are expressed as mg QE/g extract.

#### 4.6.3. Determination of the Total Carotenoid Content (TCC)

The total carotenoid content (TCC) in the KBD extract was quantified by UV-visible spectrophotometry, according to a previously established protocol [22]. In summary, the KBD extract underwent preliminary treatment with acetone and hexane. Subsequently, 100 µL of these prepared extract solutions were placed into 96-well plates, and absorbance readings were recorded at 450 nm using a microplate reader. The experiment was performed in triplicate and the results are expressed as mg β-CE/g extract.

#### 4.6.4. Determination of the Total Anthocyanin Content (TAC)

The TAC in the KBD extract was determined based on the pH differential method with a slight modification [22]. Briefly, 100 μL of 0.025 M potassium chloride solution (pH = 1) was added to 20 μL of the extracts. Then, 20 μL of the extracts were diluted with 0.4 M sodium acetate solution (pH = 4.5) in the same dilution factor. The absorbance was measured at 535 and 700 nm using a microplate reader. The experiments were performed in triplicate and the results are expressed as mg cyanidin-3-glucoside equivalents/g extract (mg C3G/g extract).

### 4.7. Statistical Analysis

The BBD, response surface analysis, and ANOVA were performed using Design-Expert software (Version 13, Stat-Ease Inc., Minneapolis, USA) to design the experiments and to evaluate the effectiveness of the variable conditions on the percentage extraction yield and total active content (phenolic, flavonoid, carotenoid, and anthocyanin). The significant results were evaluated at the confidence level of 95% (*p*-value ≤ 0.05).

## 5. Conclusions

The KBD formula is currently commercialized as powder-filled capsules. In order to improve its efficiency, other pharmaceutical formulations are needed. For this purpose, the extract of KBD powder is necessary. Among various methods of extraction, the SFE method was demonstrated to be the most effective and eco-friendly technique for extraction of bioactive natural products such as flavonoids, phenolic compounds, and carotenoids, despite the total percentage yield being inferior to that obtained by conventional SLE as well as by UAE and MAE. The fact that ScCO_2_ used in SFE is non-toxic, more economical, easily accessible, and separable from the extracts makes this technique a suitable green technology. Although ScCO_2_ presents some limitations such as its inefficiency in extracting more polar compounds such as anthocyanins, this shortcoming could be overcome by adding a small quantity of a more polar solvent such as ethanol as a co-solvent. Therefore, SFE could be the efficient process for the extraction of active compounds, especially flavonoids and carotenoids from KBD powder. The optimization of the variables of the extraction techniques by the BBD, with the aid of RMS, provides the advantage for the selection of extraction conditions. The findings from this study could be useful to obtain a superior extract for the development of new KBD formulations from the KBD powder with higher concentrations of active compounds.

## Figures and Tables

**Table 1:** BBD matrix for percentage yield, TPC, TFC, and TCC from KBD powder by SFE.

Run Order	Extraction Variables			Response							
	Temperature (°C)	Pressure (bar)	Time (min)	Percentage Extraction Yield (%)		TPC (mg GAE/g Extract)		TFC (mg QE/g Extract)		TCC (mg β-CE/g Extract)	
				Experimental	Predicted	Experimental	Predicted	Experimental	Predicted	Experimental	Predicted
1	−1 (30)	0 (250)	1 (90)	3.28	3.31	138.78	102.31	206.30	209.48	2.99	2.86
2	0 (45)	0 (250)	0 (60)	3.26	3.49	135.33	155.38	164.97	165.46	9.39	10.74
3	0 (45)	0 (250)	0 (60)	3.51	3.49	130.45	155.38	167.97	165.46	10.95	10.74
4	0 (45)	1 (300)	1 (90)	3.53	3.46	131.45	176.63	175.08	181.82	10.33	10.01
5	−1 (30)	1 (300)	0 (60)	3.57	3.61	134.78	126.07	190.63	180.72	10.5	10.95
6	0 (45)	0 (250)	0 (60)	3.39	3.49	145.36	155.38	159.41	165.46	11.34	10.74
7	−1 (30)	0 (250)	−1 (30)	3.05	3.04	127.22	166.00	189.08	197.53	12.55	12.17
8	−1 (30)	−1 (200)	0 (60)	3.08	3.01	125.22	131.62	170.37	168.66	7.70	7.76
9	0 (45)	−1 (200)	−1 (30)	2.67	2.74	82.33	37.15	148.63	141.89	4.94	5.26
10	1 (60)	−1 (200)	0 (60)	3.48	3.44	237.67	246.38	142.08	151.99	17.13	16.68
11	1 (60)	1 (300)	0 (60)	3.81	3.88	327.22	320.82	183.08	184.79	18.56	18.50
12	1 (60)	0 (250)	−1 (30)	3.50	3.47	115.56	152.03	188.86	185.68	8.72	8.85
13	0 (45)	0 (250)	0 (60)	3.50	3.49	233.44	155.38	171.63	165.46	10.81	10.74
14	1 (60)	0 (250)	1 (90)	3.58	3.59	464.56	425.78	217.19	208.74	22.26	22.64
15	0 (45)	−1 (200)	1 (90)	3.13	3.16	287.78	317.85	152.97	151.51	10.19	10.26
16	0 (45)	1 (300)	−1 (30)	3.52	3.49	277.33	247.26	154.97	156.43	10.59	10.52
17	0 (45)	0 (250)	0 (60)	3.80	3.49	132.33	155.38	163.3	165.46	11.21	10.74

**Table 2:** The analysis of variance (ANOVA) results of the response surface model of percentage extraction yield, TPC, TFC, and TCC by SFE of KBD powder.

Source	DF	Percentage Extraction Yield (%)		TPC (mg GAE/g Extract)		TFC (mg QE/g Extract)		TCC (mg β-CE/g Extract)	
		F-Value	*p*-Value	F-Value	*p*-Value	F-Value	*p*-Value	F-Value	*p*-Value
Model	9	4.72	0.0265 *	5.66	0.0162 *	8.67	0.0047 *	77.70	<0.0001 *
A	1	9.03	0.0198 *	17.16	0.0043 *	1.01	0.3474	277.81	<0.0001 *
B	1	20.02	0.0029 *	0.8500	0.3872	12.89	0.0089 *	25.72	0.0014 *
C	1	2.84	0.1357	7.90	0.0261 *	7.85	0.0265 *	20.61	0.0027 *
AB	1	0.2392	0.6397	0.5730	0.4738	1.38	0.2789	0.9617	0.3594
AC	1	0.2102	0.6605	10.20	0.0152 *	0.3953	0.5495	273.41	<0.0001 *
BC	1	1.89	0.2114	11.05	0.0127 *	0.7964	0.4018	15.56	0.0056
A^2^	1	0.6959	0.4317	1.73	0.2304	31.75	0.0008 *	61.73	0.0001 *
B^2^	1	0.8501	0.3872	0.4367	0.5299	17.83	0.0039 *	0.0280	0.8718
C^2^	1	6.68	0.0363 *	0.7516	0.4147	6.10	0.0428 *	27.51	0.0012 *
R^2^		0.8584		0.8792		0.9177		0.9901	
Adj-R^2^		0.6764		0.7239		0.8119		0.9773	

* Significant at *p*-value less than 0.05.

**Table 3:** The variable factors and their levels for optimization of SFE for KBD.

Factor	Symbol	Levels		
		−1	0	1
Extraction temperature (°C)	A	30	45	60
Extraction pressure (bar)	B	200	250	300
Extraction time (min)	C	30	60	90

**Table 4:** Experimental conditions from the BBD of each extraction run for KBD powder by SFE.

Run Order	Extraction Variables		
	Extraction Temperature (°C)	Extraction Pressure (bar)	Extraction Time (min)
1	30	250	90
2	45	250	60
3	45	250	60
4	45	300	90
5	30	300	60
6	45	250	60
7	30	250	30
8	30	200	60
9	45	200	30
10	60	200	60
11	60	300	60
12	60	250	30
13	45	250	60
14	60	250	90
15	45	200	90
16	45	300	30
17	45	250	60

## Data Availability

Not applicable.

## Sample Availability

Not available.
